# Low Frequency Magnetic Fields Enhance Antitumor Immune Response against Mouse H22 Hepatocellular Carcinoma

**DOI:** 10.1371/journal.pone.0072411

**Published:** 2013-11-20

**Authors:** Yunzhong Nie, Yueqiu Chen, Yongbin Mou, Leihua Weng, Zhenjun Xu, Youwei Du, Wenmei Wang, Yayi Hou, Tingting Wang

**Affiliations:** 1 State Key Laboratory of Pharmaceutical Biotechnology, Division of Immunology, Medical School, Nanjing University, Nanjing, China; 2 Stomatological Hospital Affiliated Medical School, Nanjing University, Nanjing, China; 3 National Laboratory of Solid Microstructures, Nanjing University, Nanjing, China; 4 Jiangsu Key Laboratory of Molecular Medicine, Nanjing University, Nanjing, China; Istituto Superiore di Sanità, Italy

## Abstract

**Objective:**

Many studies have shown that magnetic fields (MF) inhibit tumor growth and influence the function of immune system. However, the effect of MF on mechanism of immunological function in tumor-bearing mice is still unclear.

**Methods:**

In this study, tumor-bearing mice were prepared by subcutaneously inoculating Balb/c mice with hepatocarcinoma cell line H22. The mice were then exposed to a low frequency MF (0.4 T, 7.5 Hz) for 30 days. Survival rate, tumor growth and the innate and adaptive immune parameters were measured.

**Results:**

MF treatment could prolong survival time (n = 28, p<0.05) and inhibit tumor growth (n = 9, p<0.01) in tumor-bearing mice. Moreover, this MF suppressed tumor-induced production of cytokines including interleukin-6 (IL-6), granulocyte colony- stimulating factor (G-CSF) and keratinocyte-derived chemokine (KC) (n = 9–10, p<0.05 or 0.01). Furthermore, MF exposure was associated with activation of macrophages and dendritic cells, enhanced profiles of CD4^+^ T and CD8^+^ T lymphocytes, the balance of Th17/Treg and reduced inhibitory function of Treg cells (n = 9–10, p<0.05 or 0.01) in the mice model.

**Conclusion:**

The inhibitory effect of MF on tumor growth was related to the improvement of immune function in the tumor-bearing mice.

## Introduction

Biological effect of magnetic fields (MF) has been widely investigated. Epidemiological studies suggested that extremely low-frequency electromagnetic field (ELF-EMF) may induce the risks of diseases, such as childhood cancer, Alzheimer’s disease and breast cancer [1], [2], [3]. However, some experimental studies indicated that there is no relationship between extremely low-frequency magnetic field (ELF-MF) and the risks of diseases in mice model [4], [5], [6]. Interestingly, substantial evidences have shown that ELF-MF possess significant antitumor activities *in vitro* and *in vivo*. Sixty Hz sinusoidal MF significantly inhibited cell growth and induced apoptosis of prostate cancer cells by cleaving caspase-3 and accumulating reactive oxygen species [7]. Oxygen radicals could be accumulated in K562 cells under exposure to 50 Hz ELF-MF (0.025 mT to 0.1 mT) [8]. ELF-MF (0.5–16.5 Hz) combined with a static MF markedly suppressed tumor growth in Ehrlich ascites carcinoma model mice [9]. Our previous study also demonstrated that a 0.4 T, 7.5 Hz MF inhibited tumor cell growth by distributing the cell cycle [10]. To date, several hypotheses were advanced to explain the antitumor mechanism of MF, including accumulated reactive oxygen species (ROS) [11], modified cell membrane [12] and increased calcium signaling [13]. However, these hypotheses on the antitumor activity of MF mainly based on the experimental results from cell lines *in vitro* without regard to immune system of organism.

As is well known, immune surveillance of immune system in organism plays essential roles in the prevention of tumors, i.e., protecting the host from virus-induced tumors, preventing the establishment of an inflammatory environment conducive to tumorigenesis and eliminating tumor cells [14]. Of note, immune surveillance is not always successful, tumors can escape immune surveillance and ‘edit’ immune system to hamper antitumor adaptive and innate immune responses and even promote tumor progression [15], [16]. Thus it is necessary to understand whether MF changes the immunologic surveillance in tumor-bearing mice. Fortunately, some roles of MF on immune system had been reported [17], [18]. Phagocyte activity, ROS release and interleukin-1β (IL-1β) production of mouse macrophages were significantly promoted after continuous exposure to 50 Hz ELF-MF (1mT) [19]. ELF-MF (0.97 mT, 50 Hz) could alter hematologic variations, eosinophil, hemoglobin and mean platelet volume (MPV) in rats [20]. Nature Killer cell (NK) activity in workers negatively correlated with exposure to different levels of ELF-MF (Time-Weighted Average (TWA) ≤0.2 µT and TWA >0.2 µT) [21]. It is thus evident that MF may modify the immune surveillance of immune system. However, it is still need to clarify the detailed effect of MF on subpopulation of lymphocytes and their secreted cytokine levels in immune system of tumor-bearing mice.

Therefore, in our present study, we explored the correlation between tumor development and immunity in tumor-bearing mice after exposure to the effective MF (0.4 T, 7.5 Hz), which has been shown to inhibit the growth of cancer cells *in vitro* [10]. The effects of MF on innate and adaptive immune parameters, including macrophage, dendritic cell, CD4^+^ T and CD8^+^ T lymphocytes in tumor-bearing mice were analyzed. Furthermore, in order to investigate the effects of MF on subpopulation of T lymphocytes, mRNA expressions of transcription factors for Th1, Th2, Th17 and Treg, i.e., T-bet, GATA binding protein 3 (GATA3), RAR-related orphan receptor γ (RORγ), and Forkhead box protein 3 (Foxp3), respectively, were also detected.

## Results

### 2.1 Magnetic Fields Inhibit Tumor Growth

**Figure 1:**
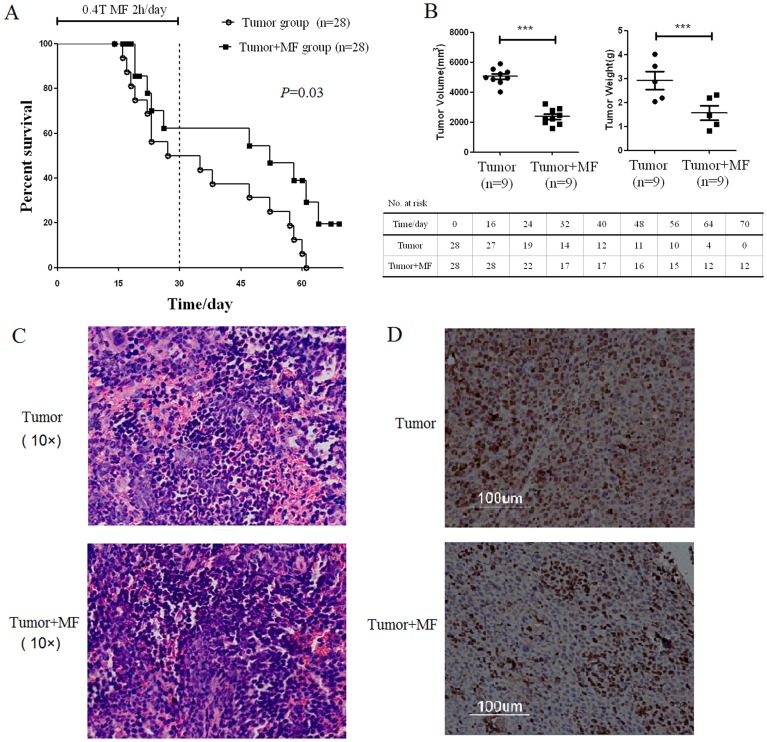
Magnetic field exposure system.

We first examined the biological effects of low-frequency MF on the tumor-bearing mice. The survival rate of mice in each group was calculated after exposure to MF for 30 days and recovery for 40 days. The results showed that there was no significant difference of mortality in the control and control+MF groups during exposure to MF for 30 days. However, by the end of the experiment, the mortality of mice in the tumor group reached 100%, while the survival rate of mice in tumor+MF group was 19.5% (n = 28, p<0.05) (Fig. 1A). It is thus evident that there was a significant different mortality between tumor group and tumor+MF group (*P* = 0.03) using log-rank test.

When tumors were removed and measured after exposure to MF for 30 days, it was showed that tumor growth (including tumor volume and tumor weight) was significantly inhibited in tumor +MF group compared with that in tumor group (n = 9, p<0.01) (Fig. 1B). Hematoxylin and eosin staining showed that more intratumoral leukocyte populations were found in tumor group than that in tumor+MF group. Immunohistochemical staining of Ki67, which presents proliferating cells, showed that Ki67+ cells were found more in tumor group than that in tumor+MF group (Fig. 1C and 1D). These data together suggest that MF exposure may inhibit tumor proliferation and prolong survival of **tumor-bearing mice**.

### 2.2 Magnetic Fields Reverse Levels of Cytokines in Plasma of Tumor-bearing Mice

**Figure 2:**
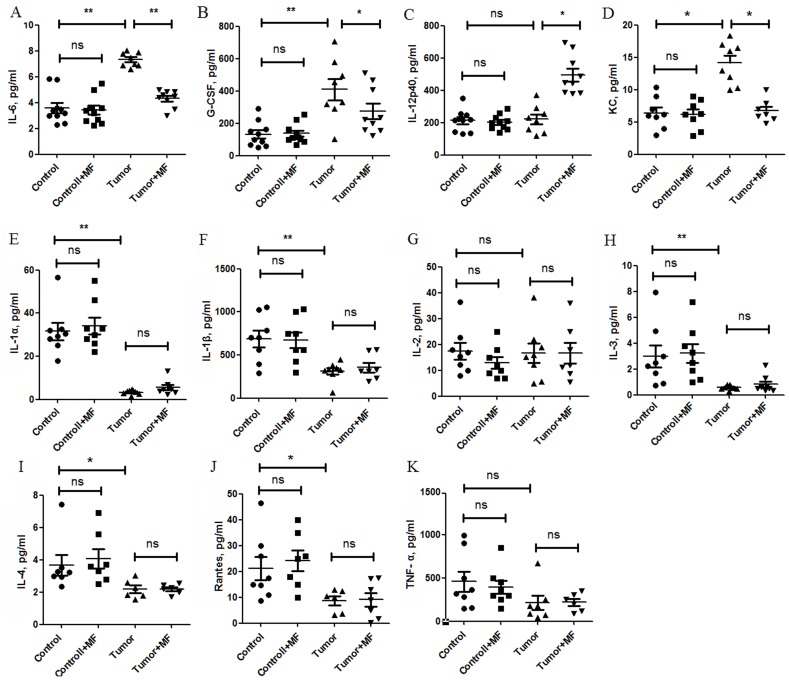
Magnetic fields raise survival rate and inhibit tumor growth in tumor-bearing mice. (A) Statistical analyses of survival after subcutaneous challenge of Balb/c mice with H22 cells and treated with magnetic fields using log-rank test. Mice were treated by magnetic fields (MF) for 30 days, 2 h per day, and cared without magnetic fields for another 40 days. (B) The mean mass and volume of tumor for each group after treatment with magnetic fields 30 days. ***P<0.001, Mann-Whitney U test. The Hematoxylin and eosin staining (C, 10×) and immunohistochemical staining of Ki67 (D) in tumors.

To test the effects of MF on cytokines, the levels of 27 cytokines were quantitated in the plasma panels by cytokine assay. Compared with control group, IL-6 and G-CSF were elevated about 2-fold in tumor group (Fig. 2A, B). After exposure to MF for 30 days, levels of IL-6 and G-CSF in tumor+MF group were reverted to those in control group (Fig. 2A, B). Moreover, IL-12, an antitumor cytokine [22], was significantly increased in tumor +MF group (Fig. 2C). However, keratinocyte-derived chemokine (KC), an important chemokine involved in the proliferation and metastasis of tumor, was increased in tumor group, while it was declined in tumor +MF group (Fig. 2D). For other cytokines, including IL-1, IL-2, IL-3, IL-4, Rantes and TNF-α (Fig. 2E–K), significant changes were not observed between tumor group and tumor+MF group.

### 2.3 Effect of Magnetic Fields on Innate Immune Cells

**Figure 3:**
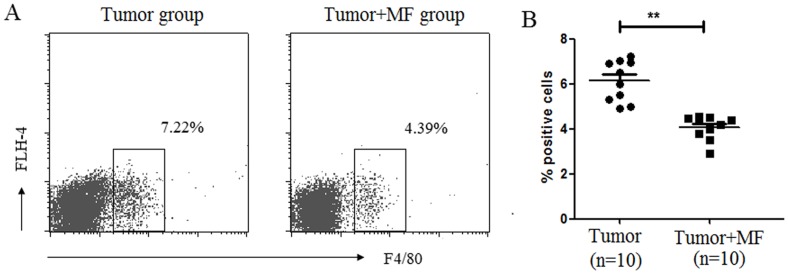
Magnetic fields reverse levels of cytokines/chemokines in plasma of tumor-bearing mice. Bio-Plex was used to analyze cytokine/chemokine changes in mice plasma, (A) IL-6, (B) granulocyte colony-stimulating factor (G-CSF), (C) IL-12p40, (D) Keratinocyte-derived Chemokine (KC), (E) IL-1α, (F) IL-1β, (G) IL-2, (H) IL-3, (I)IL-4, (J)Rantes, and (K) TNF-α (±s.e.m.). *P<0.05, **P<0.01, or ns = No significant difference.

**Figure 4:**
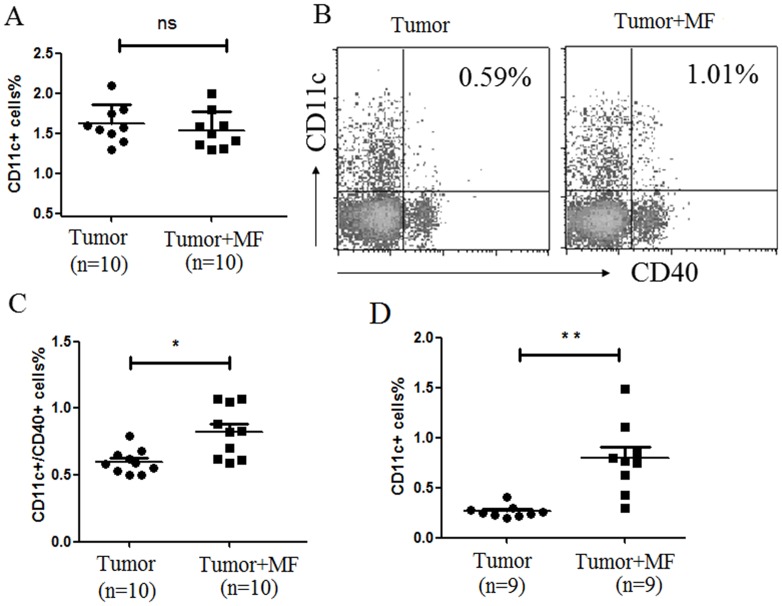
Magnetic fields transform the development of macrophage subsets. (A) Proportion of F4/80^+^ macrophage cells in PBMC was detected by flow cytometry. (B) The mean proportion of F4/80^+^ macrophage cells in PBMC for each group (Mean ± s.e.m.). **P<0.01.

The above experiment showed that MF elevated IL-12 levels in the plasma of tumor-bearing mice. Since IL-12 is one of the special cytokines secreted by macrophage, so macrophage in peripheral blood mononuclear cell (PBMC) was analyzed. The results showed that MF treatment decreased the number of F4/80^+^ macrophages in **tumor-bearing mice** (n = 10, p<0.01) (Fig. 3A, B). In addition, the change of dendritic cell (DC) was tested after exposure to MF. The results showed that the profile of DC characterized by CD11c^+^ expression in PBMC had no difference in these groups (Fig. 4A). However, after exposure to MF, both the population of CD11c^+^CD40^+^ DC (n = 10, p<0.05) (Fig. 4B,C) and tumor infiltrating CD11c^+^ DC (n = 9, p<0.01) (Fig. 4D) were significantly increased. These data indicated that MF may change the distribution of innate immune cells in tissues.

### 2.4 Effect of Magnetic Fields on T Lymphocytes

**Figure 5:**
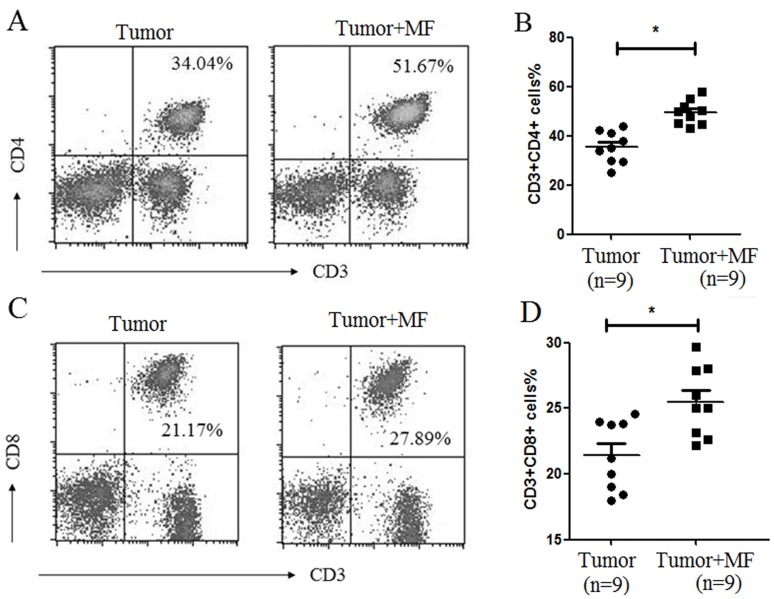
Magnetic fields enhances the expression of CD40 in Dendritic Cell. Proportion of CD11c^+^ DC (A) and CD11c^+^CD40^+^ DC (B) in PBMC was detected by flow cytometry. (C) The mean proportion of CD11c^+^CD40^+^ DC in PBMC for each group (Mean ± s.e.m.). (D) The mean proportion of CD11c^+^CD40^+^ DC in tumor infiltrating lymphocytes for each group (Mean ± s.e.m.). *P<0.05, **P<0.01 or ns = No significant difference.

We then analyzed the profiles of T lymphocytes in PBMC. As shown in Fig. 5A and 5B, CD3**^+^**CD4**^+^** T lymphocytes and CD3**^+^**CD8**^+^** T lymphocytes populations were higher in tumor+MF group than those in tumor group (n = 9, p<0.05). These results suggest that MF may promote the development of T lymphocytes.

**Figure 6:**
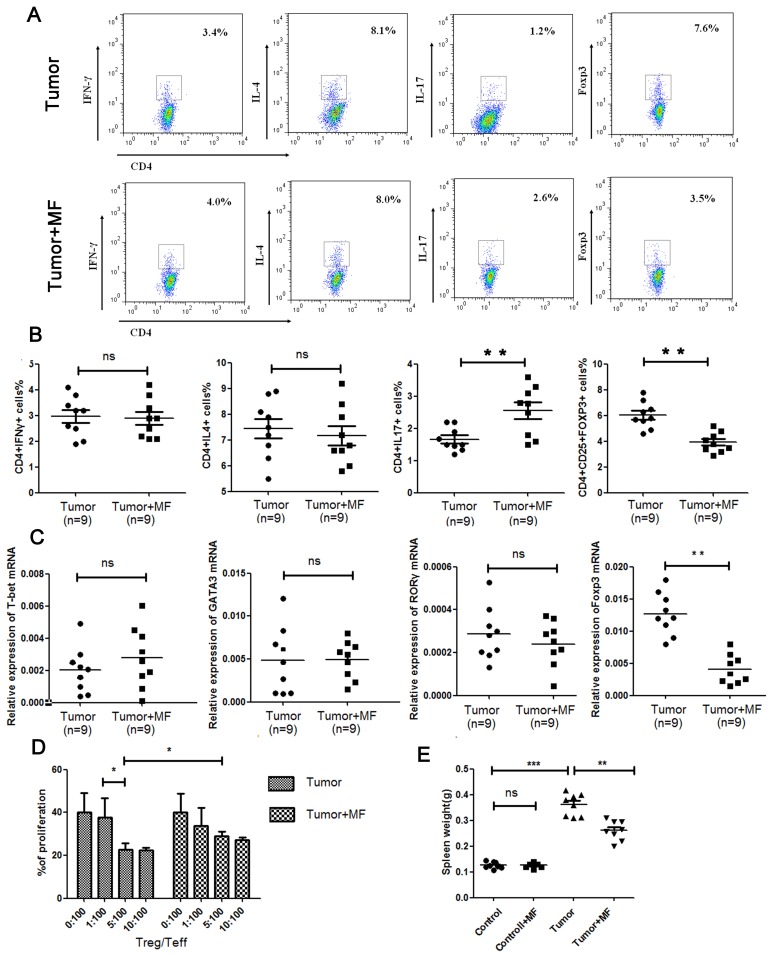
Magnetic fields promotes the development of CD4^+^ and CD8^+^ T cells polarization in PBMC. (A) Proportion of CD4^+^ T cells in PBMC was detected by flow cytometry. (B) The mean proportion of CD4^+^ T cells in PBMC for each group (Mean ± s.e.m.). (C) Proportion of CD8^+^ T cells in PBMC was detected by flow cytometry. (D) The mean proportion of CD8^+^ T cells in PBMC for each group (Mean ± s.e.m.). *P<0.05.

Usually, CD4^+^ Th cells can be roughly classified into Th1, Th2, Th17 and Regulatory T cells (Treg cells). We thus further analyzed the effect of MF on the distribution of T cell subsets from mouse spleen. As shown in Fig. 6A and 6B, MF increased Th17 cell subpopulation but decreased Treg cell subpopulation (n = 9, p<0.01). By measuring mRNA expression levels of the given transcription factors of T lymphocyte, T-bet, GATA3, RORγ and Foxp3, the results showed that Foxp3 mRNA expression levels indeed was decreased (n = 9, p<0.01), but RORγ expression was too little to detect its change (Fig. 6C). These results suggest that MF may regulate Th17/Treg balance to shift toward a Th17-type and inhibit development of Treg cells in spleen. As has been reported, Treg cells can block the immune surveillance during tumorigenesis by inhibit the function of effective T cells (Teff). Furthermore, we found that MF down-regulated the inhibitory function of Treg cells on Teff (Fig. 6D), suggesting MF may promote the anti-tumor effect of immune system through reducing Treg. In addition, the spleen plays an important role in active immune response through humoral and cell-mediated pathways. As shown in Fig. 6E, enlarged spleen was observed in **tumor-bearing mice**, which may be exerted by H22 tumor cells, while this enlargement could be reduced by MF.

## Discussion

In the recent decades, the biological effects of MF have been widely investigated. Several hypotheses were advanced to explain the molecular mechanism of MF on cancer. **However, these hypotheses on the antitumor activity of MF mainly based on the experimental results from cell lines** ***in vitro.*** In the present study, we showed that survival time of mice was prolonged and the growth of tumors was inhibited by 7.5 Hz MF at a flux density of 0.4 T. When the levels of cytokines and the distribution of immune cells in **tumor-bearing mice** were detected, we found that MF could change the function and distribution of both innate immune cells and adaptive immune cells. Especially, MF could alter the population and function of T subsets in spleen. These data suggest that MF may inhibit tumor growth through improving immune function in the tumor-bearing mice.

Our previous study had demonstrated that the proliferation of cancer cells could be inhibited by 7.5 Hz MF at a flux density of 0.4 T *in vitro* [10]. Consistently, we here found that tumor growth was also inhibited and the survival rate was elevated after exposure to this MF *in vivo*. Similar result also had been reported in mice inoculated with the Ehrlich ascites carcinoma [9]. It is thus evident that the MF (0.4 T, 7.5 Hz) indeed could inhibit tumor growth *in vitro* and *in vivo,* which suggest that the MF may be useful to treat patients with some cancers.

Immune cells may exert their functions through secretion of cytokines. Therefore, we first detected the effect of on cytokine secretion. Usually, IL-6 and G-CSF are known as pro-inflammatory cytokines. IL-6 signaling can function as a regulator of tumor cell proliferation in cancer [23], [24], while G-CSF is thought to promote tumor angiogenesis via increasing circulating endothelial progenitor cells and Gr1^+^CD11b^+^ cells in cancer animal models [25]. Moreover, IL-12 is an anti-tumor cytokine and mediates enhancement of the cytotoxic activity of NK cells and CD8^+^ cytotoxic T lymphocytes to eliminate cancer cell [26]. In addition, chemokines such as KC was involved in the processes of angiogenesis and tumorigenesis [27]. In the tumor-bearing mice, the levels of IL-6, G-CSF and KC were elevated. Strikingly, after exposure of the tumor-bearing mice to MF, the levels of IL-6, G-CSF and KC were decreased, while IL-12 was increased. These results indicated that the MF can regulate the expression of some cytokines and chemokines so as to inhibit the proliferation and tumorigenesis in tumor-bearing mice.

It has been found that the functions of macrophages can be changed in 1 mT MF for 45 min [28]. It has been known that macrophage can be divided into M1- and M2- like macrophage. M1-like macrophage can help to eliminate and M2-like macrophage can be beneficial to the tumor growth [29]. IL-12 is a specific cytokine secreted by M1-like macrophage [30], and the expression level of IL-12 is associated with the percentage of M1-like macrophage in F4/80+ macrophage. In tumor group, the percentage of F4/80+macrophage was increased, while the level of IL-12 was equal to the normal mice. So we presume that the increased macrophage may be the M2-like macrophage in tumor group. However, after exposure to MF, the percentage of macrophage was decreased, while the expression of IL-12 was increased. So the increased macrophage might be the M1-like macrophage. These findings suggested that the MF may induce the differentiation of macrophage toward M1-like macrophage to eliminate tumor.

MF enhanced the expression of CD40 in DC. CD40 is a co-stimulatory molecule and can activate CD4^+^ T lymphocytes via CD40-CD40L interaction, and help the cytotoxicity of CD8^+^ T lymphocytes [31]. Bistolfi had found that MF can induce microvilli carpeted in the plasma membrane of several types of cells including DC [32]. The carpeted microvilli had been exhibited to connect DC-T cell interaction [33]. Although little known about the relation between microvilli and CD40, but these studies suggest that MF may enhance DC-T cell interaction to eliminate cancer cell.

MF could increased percentage of T lymphocytes. In tumor-bearing mice there was an obviously high proportion of CD4^+^ T and CD8^+^ T cells after exposure to MF. However, Salerno et al., [34] found that MF can transiently decrease CD4^+^T cell proliferation *in vitro*. We hypothesized the increasing proportion of CD4^+^T cell may be attributed to the recruitment of M1-like macrophage and CD11c^+^CD40^+^ DC for CD4^+^T cell *in vivo,* which needs further research to prove.

Although these accumulated data proved that MF could activate the innate and adaptive immune, but it is yet needs to further explore about the biological effects of MF on immnue system. It is possible that MF may affect the differentiation and polarization of immune cells and the secretion of cytokines through regulating their relative gene expressions. it is also possible that MF may change microRNA profile, which is thought to regulte the function of many cells including immune cells. In addition, the effects of MF on the immune system of different trait mice also needs to be clarified. Normal mice, node mice and SCID (severe combind immuno deficiency) mice should be used to explore the detailed target of MF, since they represent normal immune system, T-cell function deficient condition and T/B function combined deficient condition, respectively.

In conclusion, our present study demonstrates that low-frequency MF could raise the survival rate and inhibite tumor growth in tumor-bearing mice. Moreover, the MF suppressed tumor-induced production of IL-6, G-CSF and KC, and enhanced the level of IL-12. Furthermore, MF exposure was associated with activation of macrophages and dendritic cells, enhanced profiles of CD4^+^ T and CD8^+^ T lymphocytes, the balance of Th17/Treg and reduced inhibitory function of Treg cells in tumor-bearing mice. Taken together, these results suggest that the inhibitory effect of MF on tumor growth may be attributed to the improvement of immune function in the tumor-bearing mice and magnetotherapy may be a potential anti-cancer therapy.

## Materials and Methods

### 4.1 Ethics Statement

This study was carried out strictly accordance with the recommendations in the Guide for the Care and Use of Laboratory Animals of the National Institutes of Health. All efforts were made to minimize suffering. The protocol was approved by the Committee on the Ethics of Animal Experiments of Drum Tower Hospital ethics committee (No. 2012113105, Date 2012/3/2).

### 4.2 Experimental Magnetic Fields

**Figure 7:**
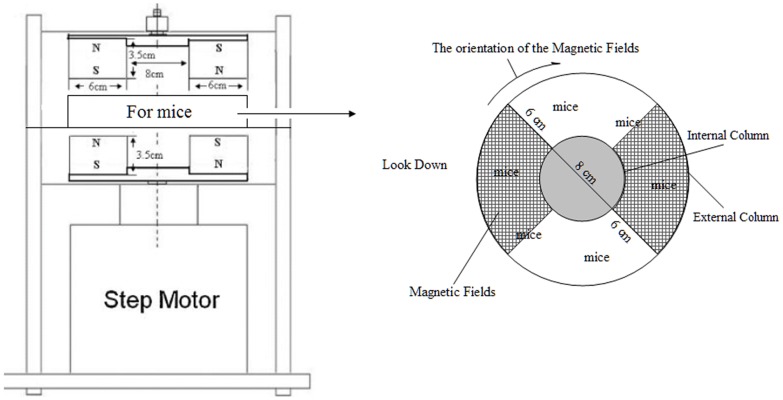
Magnetic fields regulate Th17/Treg balance and down-regulate the inhibitory function of Treg cells in spleen. (A) Flow cytometry analysis of Th1, Th2, Th17 and Treg cell populations in spleen. (B) Summarized data of Th1, Th2, Th17 and Treg cell populations in spleen. (C) mRNA expression of T-bet, GATA3, RORγ and Foxp3 in splenic lymphocytes were detected by qPCR. (D) Inhibitory function of Treg cells in each group were detected by mixed lymphocyte reaction. (E) The mean mass of spleen for each group (Mean ± s.e.m.). *P<0.05, **P<0.01, ***P<0.001, or ns = No significant difference.

The construction of experimental magnetic fields has been described previously [10]. Briefly, two pairs of fan-shaped NdFeB permanent magnets (N45, Innuovo, Dongyang, China) were embedded into a circular iron plate and arranged to establish MF. The bottom two magnets rotated at certain frequency driven by a step motor, which was controlled using a functional signal generator. The top two magnets rotated synchronously due to the strong magnetic interaction. Magnetic flux density was measured at the target site using a gauss meter (HT201, Hengtong, Shanghai, China). MF at the target site is alternative pulses with a maximum flux density of about 0.4 T and a fixed frequency of 7.5 Hz. As shown in Fig. 7, mice were placed in the middle of the magnets in a lucent and breathable box. There was an internal column in the center of the box, and mice were put between external column (D = 20 cm) and internal column (D = 8 cm). Mice can move freely in the box. Ten mice can simultaneously be exposed in this MF exposure set-up. The magnetic field was rotated clockwise. This instrument was fabricated by the National Laboratory of Solid Microstructures, Nanjing University (Nanjing, China). Control mice were placed in a similar apparatus except that there were two rotating iron plates instead of magnets, thus lacking a MF, sham MF.

### 4.3 Animals and Experimental Groups

4–6 week-old female Balb/c mice from same parents were purchased from the Animal Research Center of Yangzhou University, PR China. Mice were maintained under specific pathogen-free conditions in a temperature-controlled room (24°C), on a 12-h/12-h light and dark cycle. Standard laboratory pelleted formula and tap water were provided. Each group of mice was housed in separate rack-mounted wire cages. All the cages were placed in the same shelf. A quick death procedure by cervical dislocation was uniformly performed in all animals. The experiments were conducted according to institutional animal ethics guidelines. All efforts were made to minimize suffering.

For construct the implanting tumor mice model, 1×10^6^ H22 hepatocarcinoma cells were subcutaneously injected into the armpit region of 6–8 week-old Balb/c mice. At the beginning, Mice (n = 96) were randomly divided into four groups: Control group (n = 10), normal mice exposed to sham MF; Control+MF group (n = 10), normal mice exposed to MF; Tumor group (n = 38), the mice were subcutaneously injected with H22 hepatocarcinoma cells and exposed to sham MF; Tumor+MF group (n = 38), the mice were subcutaneously injected with H22 hepatocarcinoma cells and exposed to MF. After three days subcutaneously injected with cancer cells, mice were exposed to sham MF or MF (0.4 T, 7.5 Hz) for 30 days, the exposure time was 2 h per day. The descriptive statistics of mice were shown in Table S1. 10 mice in each group were sacrificed on day 33. Cardiac blood was collected from each mouse and centrifuged at 450×g for 20 min. Plasma was obtained and stored at −70°C until use. The tumor and spleen was removed and measured from each mouse. Part of each spleen was used to flow cytometry analysis, and others were used to isolate RNA. The remaining mice were used to observe the survival time (Tumor group n = 28, Tumor+MF group n = 28). The operators were blinded to the group of mice during all the experiments.

### 4.4 Immunohistochemistry Analysis

Tumor tissues were harvested, fixed in 10% buffered formalin, dehydrated, bisected, mounted in paraffin, and sectioned. Hydrated sections were stained using Hematoxylin/Eosin. IHC was carried out with antibodies specific for Ki67 using rabbit anti-mouse Ki67 (1∶1600, Dako Cytomation, Denmark).

### 4.5 Cytokine Assay

Serums were analyzed for cytokines using a high-sensitivity mouse cytokine assay (Bio-Rad, Hercules, CA) according to manufacturer’s instructions. Briefly, 50 µl of cytokine standards or samples were incubated with 50 µl of anti-cytokine conjugated beads in 96-well filter plates for 30 min at room temperature with shaking. Plates were then washed by vacuum filtration three times with 100 µl wash buffer, 25 µl of diluted detection biotinylated antibody was added, and plates were incubated for 30 min at 25°C with shaking. After washing for three times, 50 µl of streptavidin-phycoerythrin was added, and the plates were incubated for 10 min at room temperature with shaking. Finally, plates were washed by vacuum filtration three times, and beads were suspended in 125 µl of assay buffer. Data were acquired using a Luminex system (Luminex-100, Bio-Rad) and analyzed using Manager software (V4.1, Bio-Rad). The minimum detection concentration was 0.2 pg/ml. Each sample was assayed in duplicate, and cytokine standards supplied by the manufacturer were run on each plate. In all cases tested, comparable elevations were observed using the Luminex-based multiplex assays. The cytokine assay allows detecting the following cytokines and chemokines: IL-1α, IL-1β, IL-2, IL-3, IL-4, IL-5, IL-6, IL-9, IL-10, IL-12(p40), IL-12(p70), IL-13, IL-17, G-CSF, GM-CSF, IFN-γ, KC, MCP-1, MIP-1α, MIP-1β, Rantes and TNF-α.

### 4.6 Flow Cytometry

Peripheral Blood Mononuclear Cells (PBMC) were collected from mice and isolated from heparinized peripheral blood of the studied subjects by standard FicollePaque (General Electric Healthcare, Uppsala, Sweden) density centrifugation.

For the collection of splenic cells, single-cell suspension were dissociated by gently pressing the organ through a fine, 50 µm-nylon mesh, and cells were collected by centrifugation at 300×g for 5 min. Erythrocytes were removed by treating the splenic cells with red blood cell lysis buffer (0.15 M NH_4_Cl, 1.0 mM KHCO_3_, 0.1 mM Ethylene Diamine Tetraacetie Acid (EDTA), pH 7.2) for 5 min and washing twice with cold phosphate buffer saline (PBS).

For immune cells detection, 1×10^6^ cell were suspended in PBS and incubated with related anti-mouse antibodies for 30 min at 4°C, then washed twice with fluorescence activating cell sorter (FACS) washing buffer. Data were acquired on FACS Vantage SE (FACSCalibur, Becton Dickinson, San Jose, CA) and analyzed with CellQuest software (CellQuest Pro, Becton Dickinson). The following antibodies used for flow cytometry: anti-F4/80-PE, anti-CD3-PE-Cy5.5, anti-CD4-FITC, anti-CD8-PE, anti-CD11c-PE, anti-CD40-APC, anti-IFNγ-APC, anti-IL4-APC, anti-IL17-APC, anti-CD25-PE, anti-Foxp3-APC and anti-isotype-specific control Anti-bodies were purchased from eBioscience (eBioscience, San Diego, CA).

### 4.7 Quantitative Real Time Polymerase Chain Reaction (Q-PCR)

Total RNA was isolated using Trizol reagent (Invitrogen, Carlsbad, CA) according to the manufacturer’s instructions. A total of 1 µg RNA was used as the template for single strand cDNA synthesis utilizing random primers and the Primescript reverse transcriptase (M-MLV, Takara, Shiga, Japan) according to the manufacturer’s instructions. Q-PCR was performed for target genes. The primers used in this study were list in Table 1. The cDNA was amplified using SYBR green PCR Mix (iTAP, Bio-Rad) on a step-one plus sequence detection system (Applied Biosystems, Foster City, CA), programmed for 95°C for 10 min, then 40 cycles of 95°C for 15 s, 60°C for 30 s, and 72°C for 30 s. For the dissociation curve, reactions were incubated at 95°C for 1 min, and ramp up from 55°C to 95°C with a heating rate of 0.1°C/sec and continuous fluorescence measurement. Relative gene expression quantifications were calculated according to the comparative Ct method using β-actin as an internal standard and commercial RNA (Clontech) as calibrators. The stability ofβ-actin was confirmed in preliminary experiments. Gene expression was analysed with the StepOne software and quantified by the formula 2^−ΔΔCt^.

**Table 1:** The name and primer sequences used in real-time PCR.

Gene name	Primers (5′ to 3′)
T-bet	FP: GCCTGGCGAGTTCTTCC
	RP: GCCGTCCTTGCTTAGTGAT
GATA3	FP: GAAGGCATCCAGACCCGAAAC
	RP: ACCCATGGCGGTGACCATGC
RORγ	FP: AGATTGCCCTCTACACG
	RP: GGCTTGGACCACGATG
β-Actin	FP: GCGTGACATCAAAGAGAAGCT
	RP: ATGCCACAGGATTCCATACC

GATA3, GATA binding protein 3; ROR, retinoid-related orphan receptor; FP, forward primer;RP, reverse primer.

### 4.8 Mixed Lymphocyte Reaction (MLRs)

Treg cells were obtained using magnetic isolation kit (Miltenyi Biotec, Germany) according to the instructions. The others cells were used as effective cells (Teff). Teff were stimulated with phytohemagglutinin (5 ug/ml) and were seeded in 96-well flat-bottom plates (Corning, Life Sciences) with 1640 medium supplemented with 5% FBS, and 20 IU/mL IL2. Treg cells treated with mitomycin C were added into coculture wells with the concentration of 1∶100 (1×10^3^ Treg cells), 5∶100(5×10^3^ Treg cells) and 10∶100 (10×10^3^ Treg cells), respectively. Cell proliferation was measured by ^3^H-thymidine incorporation after the coculture of three days. Each experiment was repeated three times.

### 4.9 Statistical Evaluation

Values were expressed as mean ± SEM. Statistical analysis was performed using Mann-Whitney U test to compare the mean values between two groups. In case of survival curve, the data were analyzed by the log-rank test. Values of *P*<0.05 were considered to be statistically significant. Statistical analysis was done using the SPSS 11.5 software program (SPSS, Chicago, IL).

## Supporting Information

Table S1 **Descriptive statistics of mice.**
(DOC)
