# Supplementary material for: Low Frequency Magnetic Fields Enhance Antitumor Immune Response against Mouse H22 Hepatocellular Carcinoma
Source: PLoS One. 2013 Nov 20;8(11):e72411. doi: 10.1371/journal.pone.0072411 (PMC3835892; doi:10.1371/journal.pone.0072411)
Supplement: Table S1 — Descriptive statistics of mice. (DOC) [file pone.0072411.s001.doc]

Table S1 Descriptive statistics of mice.

|  | Control group | Control+MF group | Tumor group | Tumor+MF group |
| --- | --- | --- | --- | --- |
| Age | 7 weeks | 7 weeks | 7 weeks | 7 weeks |
| Numble | 10 | 10 | 38 | 38 |
| Sex |  |  |  |  |
| female | 10 | 10 | 38 | 38 |
| male | 0 | 0 | 0 | 0 |
| Tumor weight  (g/at death time) |  |  | 2.96  (0.78g-4.3g) | 1.62  (0.5g-2.2g) |
| Tumor size  (mm^3^/at death time) |  |  | 5020  (3960-5880) | 2198  (1850-3600) |
| Mice weight  (g/at death time) | 19.5  (17.6-22.4) | 20.6  (17.2-22.1) | 21.1  (18.2-24.6) | 20.2  (18.8-25.3) |
| Behavioural changes of mice | Not found | Not found | Not found | Not found |
| Loss of appetite | Not found | Not found | Not found | Not found |
| Hair loss | Not found | Not found | Not found | Not found |
